# 3,3′-Dihy­droxy-6,6′-bis­(hy­droxy­meth­yl)-2,2′-(pentane-1,1-di­yl)di-4*H*-pyran-4-one

**DOI:** 10.1107/S1600536812010276

**Published:** 2012-03-14

**Authors:** Mu-Song Liu, Pan-Pan Hu, Tao Zhou

**Affiliations:** a College of Pharmaceutical Science, Zhejiang University of Technology, Hangzhou, Zhejiang 310014, People’s Republic of China; b School of Food Science and Biotechnology, Zhejiang GongShang University, Hangzhou, Zhejiang 310012, People’s Republic of China

## Abstract

In the title mol­ecule, C_17_H_20_O_8_, the two pyran rings form a dihedral angle of 61.2 (2)°. The two hy­droxy­methyl groups are each disordered over two sets of sites in a 0.764 (3):0.236 (3) ratio. In the crystal, O—H⋯O hydrogen bonds link the mol­ecules into layers parallel to the *ac* plane.

## Related literature

For the biological properties of kojic acid, see: Kobayashi *et al.* (1995 ▶). For related structures, see: Nurchi *et al.* (2010 ▶); Kakkar & Singh (2011 ▶); Lokaj *et al.* (1991 ▶). For the preparation of the title compound, see: Barham & Nathan Reed (1938 ▶).
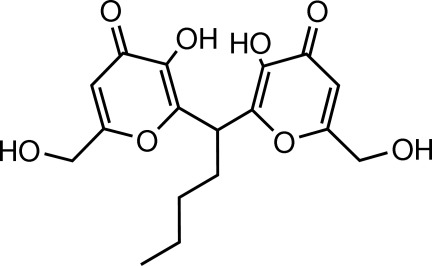


## Experimental

### 

#### Crystal data

C_17_H_20_O_8_
*M* *_r_* = 352.33
Triclinic, 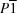

*a* = 6.4234 (3) Å
*b* = 9.2394 (4) Å
*c* = 15.8494 (7) Å
α = 79.993 (1)°
β = 86.689 (2)°
γ = 66.622 (1)°
*V* = 850.22 (7) Å^3^
*Z* = 2
Mo *K*α radiation
μ = 0.11 mm^−1^
*T* = 296 K
0.49 × 0.47 × 0.33 mm

#### Data collection

Rigaku R-AXIS RAPID/ZJUG diffractometer
Absorption correction: multi-scan (*ABSCOR*; Higashi, 1995 ▶) *T*_min_ = 0.938, *T*_max_ = 0.965
8442 measured reflections
3840 independent reflections
2907 reflections with *I* > 2σ(*I*)
*R*_int_ = 0.017

#### Refinement

*R*[*F* ^2^ > 2σ(*F* ^2^)] = 0.043
*wR*(*F* ^2^) = 0.120
*S* = 1.00
3840 reflections
251 parameters
6 restraints
H-atom parameters constrained
Δρ_max_ = 0.30 e Å^−3^
Δρ_min_ = −0.30 e Å^−3^

### 

Data collection: *PROCESS-AUTO* (Rigaku, 2006 ▶); cell refinement: *PROCESS-AUTO*; data reduction: *CrystalStructure* (Rigaku, 2007 ▶); program(s) used to solve structure: *SHELXS97* (Sheldrick, 2008 ▶); program(s) used to refine structure: *SHELXL97* (Sheldrick, 2008 ▶); molecular graphics: *ORTEP-3* (Farrugia,1997 ▶); software used to prepare material for publication: *WinGX* (Farrugia, 1999 ▶).

## Supplementary Material

Crystal structure: contains datablock(s) global, I. DOI: 10.1107/S1600536812010276/cv5255sup1.cif

Structure factors: contains datablock(s) I. DOI: 10.1107/S1600536812010276/cv5255Isup2.hkl

Supplementary material file. DOI: 10.1107/S1600536812010276/cv5255Isup3.cml

Additional supplementary materials:  crystallographic information; 3D view; checkCIF report

## Figures and Tables

**Table 1:** Hydrogen-bond geometry (Å, °)

*D*—H⋯*A*	*D*—H	H⋯*A*	*D*⋯*A*	*D*—H⋯*A*
O3—H3⋯O2^i^	0.82	1.96	2.7275 (15)	155
O3—H3⋯O2	0.82	2.32	2.7440 (15)	113
O5—H5⋯O6^ii^	0.82	2.00	2.7488 (16)	151
O5—H5⋯O6	0.82	2.32	2.7436 (16)	113
O4*A*—H4*A*⋯O2^iii^	0.82	2.07	2.839 (2)	157
O8*A*—H8*A*⋯O6^iii^	0.82	2.15	2.894 (2)	152

Symmetry codes: (i) 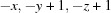; (ii) 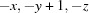; (iii) 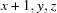.

**Fig. 1.**
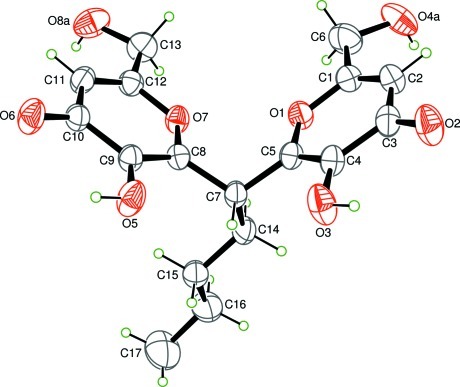
Molecular structure of (I) showing atom-labelling scheme and displacement ellipsoids at 50% probability level. Only major components of the disordered groups are shown.

**Fig. 2.**
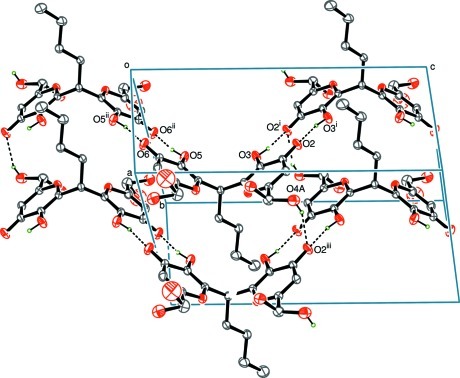
A portion of the crystal packing of (I) with hydrogen bonds shown by dashed lines [symmetry codes: (i) -x, 1 - y, -z; (ii) -x, 1 - y, 1 - z; (iii) 1 + x, y, z]. H atoms not involved in hydrogen bonding have been omitted for clarity.

## supplementary crystallographic information

### Comment

Kojic acid possesses appreciable inhibitory activity against tyrosinase, a key enzyme in the biosynthesis of melanin, due to its ability to chelate copper in the active site of this enzyme. Thus kojic acid inhibits the production of melanin pigment, consequently being used in cosmetic. Therefore, in an attempt to seek potent tyrosinase inhibitors, the derivatives of kojic acid have been widely investigated (Kobayashi *et al.*, 1995).There were little attentation to crystal structure of kojic and derivatives.The similar crystal structure of Kojic acid have been reported on 5-hydroxy-2-(hydroxymenthyl) -4*H*-pyran-one (Lokaj *et al.*, 1991; Kakkar *et al.*, 2011) and 6,6'-methylenebis (5-hydroxy-2-(hydroxymethyl)-4*H*-pyran-4-one) (Nurchi *et al.*, 2010). Herein, we report the crystal structure of the title compound (I).

In (I) (Fig. 1), two pyranone rings are planar forming the dihedral angle of 61.2 (2)°. Hydroxyl groups are almost coplanar with their linked pyranone rings forming the torsion angles O2—C3—C4—O3 of -0.10° and O6—C10—C9—O5 of 1.24°.

Intermolecular hydrogen bonds O3—H3···O2^i^ and O5—H5···O6^ii^ (Table 1) link molecules into zigzag chains along the *c* axis. Further, intermolecular hydrogen bonds O4A—H4···O2^iii^ and O8A—H8A···O6^iii^ (Table 1) link all of the components of the structure into layers parallel to *ac* plane.

### Experimental

To a solution of 5-hydroxy-(2-hydroxymethyl)-4*H*-pyran-4-one (kojic acid) (1.42 g, 10 mmol), sodium carbonate (1.06 g, 10 mmol) in water (10 ml) and methanol (10 ml) was added pentanal (10 mmol) at 343k with stirring. The stirring was continued for 3 h at that temperature. After removal of about half volume of the solvent, the soltion was neutrilized to pH=1 with concentrated hydrochloride. The crude product was obtained by filtration as an off-white solid (1.32, 75%), which was recrystallized from dichloromethane solution, giving colorless crystals of the title compound suitable for X-ray diffraction. 1H NMR (400 MHz DMSO) / d 0.84 (t, J = 6.8 Hz, 3H), 1.21 (m, 4H), 1.29 (2, m), 1.92 (m, 2H), 4.28 (s, 4H), 4.68 (t, J = 8.0 Hz, 1H), 5.62 (s, 2H), 6.28 (s, 2H), 9.03 (s, 2H).

### Refinement

H atoms were placed in calculated positions with O—H = 0.82 and C—H = 0.93–0.98 Å, and included in the refinement in riding model, with *U*_iso_(H)= 1.2 - 1.5 *U*_eq_(carrier atom). Atoms O4 and O8 of hydroxyl groups were treated as disordered over two positions - A and B, respectively - with the occupancies refined to 0.764 (3) and 0.236 (3), respectively.

### Crystal data

**Table:**

C_17_H_20_O_8_	*Z* = 2
*M**_r_* = 352.33	*F*(000) = 372
Triclinic, *P*1	*D*_x_ = 1.376 Mg m^−^^3^
Hall symbol: -P 1	Mo *K*α radiation, λ = 0.71073 Å
*a* = 6.4234 (3) Å	Cell parameters from 6451 reflections
*b* = 9.2394 (4) Å	θ = 3.3–27.4°
*c* = 15.8494 (7) Å	µ = 0.11 mm^−^^1^
α = 79.993 (1)°	*T* = 296 K
β = 86.689 (2)°	Chunk, yellow
γ = 66.622 (1)°	0.49 × 0.47 × 0.33 mm
*V* = 850.22 (7) Å^3^	

### Data collection

**Table:**

Rigaku R-AXIS RAPID/ZJUG diffractometer	3840 independent reflections
Radiation source: rotating anode	2907 reflections with *I* > 2σ(*I*)
Graphite monochromator	*R*_int_ = 0.017
Detector resolution: 10.00 pixels mm^-1^	θ_max_ = 27.4°, θ_min_ = 3.3°
ω scans	*h* = −8→8
Absorption correction: multi-scan (*ABSCOR*; Higashi, 1995)	*k* = −11→10
*T*_min_ = 0.938, *T*_max_ = 0.965	*l* = −20→20
8442 measured reflections	

### Refinement

**Table:**

Refinement on *F*^2^	Primary atom site location: structure-invariant direct methods
Least-squares matrix: full	Secondary atom site location: difference Fourier map
*R*[*F*^2^ > 2σ(*F*^2^)] = 0.043	Hydrogen site location: inferred from neighbouring sites
*wR*(*F*^2^) = 0.120	H-atom parameters constrained
*S* = 1.00	*w* = 1/[σ^2^(*F*_o_^2^) + (0.0554*P*)^2^ + 0.2871*P*] where *P* = (*F*_o_^2^ + 2*F*_c_^2^)/3
3840 reflections	(Δ/σ)_max_ < 0.001
251 parameters	Δρ_max_ = 0.30 e Å^−^^3^
6 restraints	Δρ_min_ = −0.30 e Å^−^^3^

### Special details

**Table:**

Geometry. All e.s.d.'s (except the e.s.d. in the dihedral angle between two l.s. planes) are estimated using the full covariance matrix. The cell e.s.d.'s are taken into account individually in the estimation of e.s.d.'s in distances, angles and torsion angles; correlations between e.s.d.'s in cell parameters are only used when they are defined by crystal symmetry. An approximate (isotropic) treatment of cell e.s.d.'s is used for estimating e.s.d.'s involving l.s. planes.
Refinement. Refinement of *F*^2^ against ALL reflections. The weighted *R*-factor *wR* and goodness of fit *S* are based on *F*^2^, conventional *R*-factors *R* are based on *F*, with *F* set to zero for negative *F*^2^. The threshold expression of *F*^2^ > σ(*F*^2^) is used only for calculating *R*-factors(gt) *etc*. and is not relevant to the choice of reflections for refinement. *R*-factors based on *F*^2^ are statistically about twice as large as those based on *F*, and *R*- factors based on ALL data will be even larger.

### Fractional atomic coordinates and isotropic or equivalent isotropic displacement parameters (Å^2^)

**Table:**

	*x*	*y*	*z*	*U*_iso_*/*U*_eq_	Occ. (<1)
O1	0.70599 (16)	0.37814 (12)	0.33797 (6)	0.0343 (2)	
O2	0.24351 (19)	0.37234 (15)	0.52086 (7)	0.0489 (3)	
O3	0.10419 (18)	0.56659 (15)	0.36549 (7)	0.0499 (3)	
H3	0.0303	0.5617	0.4091	0.075*	
O5	0.10800 (19)	0.56285 (16)	0.11434 (8)	0.0534 (3)	
H5	0.0366	0.5594	0.0740	0.080*	
O6	0.2475 (2)	0.35803 (17)	−0.00299 (8)	0.0562 (3)	
O7	0.70230 (17)	0.35409 (12)	0.16422 (6)	0.0381 (3)	
O4A	1.1115 (3)	0.1209 (2)	0.50117 (12)	0.0591 (5)	0.764 (3)
H4A	1.1380	0.1883	0.5209	0.089*	0.764 (3)
O8A	1.1284 (3)	0.0846 (2)	0.04825 (13)	0.0635 (6)	0.764 (3)
H8A	1.1580	0.1543	0.0169	0.095*	0.764 (3)
O4B	1.0634 (8)	0.0558 (6)	0.3693 (3)	0.0474 (14)	0.236 (3)
H4B	1.0161	−0.0084	0.3968	0.071*	0.236 (3)
O8B	1.061 (2)	−0.0030 (16)	0.0999 (9)	0.153 (5)	0.236 (3)
H8B	1.0323	0.0071	0.0490	0.230*	0.236 (3)
C1	0.7734 (2)	0.28416 (17)	0.41491 (9)	0.0348 (3)	
C2	0.6275 (3)	0.27937 (18)	0.47792 (9)	0.0375 (3)	
H2	0.6811	0.2135	0.5301	0.045*	
C3	0.3903 (2)	0.37414 (17)	0.46608 (9)	0.0347 (3)	
C4	0.3252 (2)	0.47378 (17)	0.38243 (9)	0.0329 (3)	
C5	0.4816 (2)	0.47156 (16)	0.32159 (8)	0.0302 (3)	
C6	1.0238 (3)	0.1844 (2)	0.41614 (11)	0.0474 (4)	
H6A	1.0525	0.0971	0.3848	0.057*	0.764 (3)
H6B	1.1022	0.2494	0.3873	0.057*	0.764 (3)
H6C	1.1034	0.2466	0.3877	0.057*	0.236 (3)
H6D	1.0788	0.1461	0.4755	0.057*	0.236 (3)
C7	0.4372 (2)	0.56545 (17)	0.23201 (8)	0.0331 (3)	
H7	0.2759	0.6362	0.2279	0.040*	
C8	0.4808 (2)	0.45696 (17)	0.16640 (8)	0.0332 (3)	
C9	0.3266 (3)	0.46005 (18)	0.11099 (9)	0.0363 (3)	
C10	0.3925 (3)	0.35414 (19)	0.04794 (9)	0.0400 (3)	
C11	0.6259 (3)	0.24898 (19)	0.04981 (10)	0.0437 (4)	
H11	0.6790	0.1780	0.0109	0.052*	
C12	0.7702 (3)	0.25138 (18)	0.10724 (10)	0.0407 (4)	
C13	1.0161 (3)	0.1441 (2)	0.12089 (14)	0.0593 (5)	
H13A	1.0933	0.2027	0.1416	0.071*	0.764 (3)
H13B	1.0285	0.0544	0.1654	0.071*	0.764 (3)
H13C	1.1133	0.1895	0.0883	0.071*	0.236 (3)
H13D	1.0587	0.1215	0.1812	0.071*	0.236 (3)
C14	0.5730 (3)	0.67220 (18)	0.21157 (9)	0.0396 (3)	
H14A	0.7336	0.6051	0.2178	0.047*	
H14B	0.5444	0.7244	0.1523	0.047*	
C15	0.5152 (3)	0.79877 (19)	0.26837 (10)	0.0421 (4)	
H15A	0.5501	0.7469	0.3275	0.050*	
H15B	0.3537	0.8638	0.2640	0.050*	
C16	0.6444 (4)	0.9060 (2)	0.24434 (13)	0.0565 (5)	
H16A	0.8057	0.8408	0.2504	0.068*	
H16B	0.6136	0.9542	0.1845	0.068*	
C17	0.5856 (5)	1.0365 (3)	0.29752 (16)	0.0846 (8)	
H17A	0.4309	1.1097	0.2863	0.127*	
H17B	0.6839	1.0927	0.2830	0.127*	
H17C	0.6046	0.9906	0.3572	0.127*	

### Atomic displacement parameters (Å^2^)

**Table:**

	*U*^11^	*U*^22^	*U*^33^	*U*^12^	*U*^13^	*U*^23^
O1	0.0301 (5)	0.0425 (6)	0.0298 (5)	−0.0143 (4)	0.0034 (4)	−0.0054 (4)
O2	0.0423 (6)	0.0612 (7)	0.0361 (6)	−0.0176 (6)	0.0120 (5)	−0.0004 (5)
O3	0.0323 (6)	0.0651 (8)	0.0361 (6)	−0.0075 (5)	0.0071 (4)	0.0030 (5)
O5	0.0355 (6)	0.0775 (9)	0.0462 (7)	−0.0136 (6)	−0.0004 (5)	−0.0285 (6)
O6	0.0540 (7)	0.0746 (9)	0.0452 (7)	−0.0240 (6)	−0.0054 (5)	−0.0247 (6)
O7	0.0378 (6)	0.0412 (6)	0.0340 (5)	−0.0127 (5)	0.0010 (4)	−0.0101 (4)
O4A	0.0509 (10)	0.0545 (10)	0.0667 (11)	−0.0207 (8)	−0.0180 (8)	0.0091 (8)
O8A	0.0518 (10)	0.0561 (11)	0.0838 (14)	−0.0127 (9)	0.0167 (9)	−0.0404 (10)
O4B	0.038 (3)	0.037 (3)	0.062 (3)	−0.008 (2)	0.009 (2)	−0.014 (2)
O8B	0.153 (5)	0.153 (5)	0.154 (5)	−0.060 (2)	0.0009 (10)	−0.0263 (13)
C1	0.0354 (7)	0.0357 (7)	0.0338 (7)	−0.0140 (6)	−0.0016 (6)	−0.0066 (6)
C2	0.0399 (8)	0.0384 (8)	0.0306 (7)	−0.0136 (6)	−0.0006 (6)	−0.0007 (6)
C3	0.0393 (8)	0.0364 (7)	0.0302 (7)	−0.0170 (6)	0.0054 (6)	−0.0066 (6)
C4	0.0318 (7)	0.0363 (7)	0.0301 (7)	−0.0130 (6)	0.0024 (5)	−0.0058 (6)
C5	0.0312 (7)	0.0334 (7)	0.0282 (6)	−0.0141 (6)	0.0012 (5)	−0.0071 (5)
C6	0.0351 (8)	0.0514 (9)	0.0512 (9)	−0.0134 (7)	−0.0007 (7)	−0.0052 (8)
C7	0.0350 (7)	0.0381 (7)	0.0256 (6)	−0.0136 (6)	0.0025 (5)	−0.0064 (5)
C8	0.0349 (7)	0.0377 (7)	0.0274 (6)	−0.0157 (6)	0.0044 (5)	−0.0046 (6)
C9	0.0369 (8)	0.0450 (8)	0.0283 (7)	−0.0179 (7)	0.0036 (6)	−0.0067 (6)
C10	0.0471 (9)	0.0484 (9)	0.0293 (7)	−0.0235 (7)	0.0023 (6)	−0.0082 (6)
C11	0.0541 (10)	0.0420 (8)	0.0358 (8)	−0.0171 (7)	0.0047 (7)	−0.0140 (7)
C12	0.0460 (9)	0.0372 (8)	0.0362 (8)	−0.0134 (7)	0.0047 (6)	−0.0077 (6)
C13	0.0489 (10)	0.0527 (10)	0.0658 (12)	−0.0064 (8)	0.0031 (9)	−0.0169 (9)
C14	0.0500 (9)	0.0420 (8)	0.0309 (7)	−0.0233 (7)	0.0081 (6)	−0.0063 (6)
C15	0.0520 (9)	0.0409 (8)	0.0358 (8)	−0.0207 (7)	0.0033 (7)	−0.0075 (6)
C16	0.0716 (12)	0.0488 (10)	0.0576 (11)	−0.0329 (9)	0.0096 (9)	−0.0106 (8)
C17	0.129 (2)	0.0774 (15)	0.0786 (15)	−0.0695 (16)	0.0189 (15)	−0.0289 (12)

### Geometric parameters (Å, º)

**Table:**

O1—C1	1.3502 (17)	C6—H6B	0.9700
O1—C5	1.3669 (16)	C6—H6C	0.9553
O2—C3	1.2474 (17)	C6—H6D	0.9795
O3—C4	1.3492 (17)	C7—C8	1.5069 (19)
O3—H3	0.8200	C7—C14	1.542 (2)
O5—C9	1.3518 (18)	C7—H7	0.9800
O5—H5	0.8200	C8—C9	1.350 (2)
O6—C10	1.2531 (19)	C9—C10	1.448 (2)
O7—C12	1.3529 (18)	C10—C11	1.426 (2)
O7—C8	1.3641 (17)	C11—C12	1.345 (2)
O4A—C6	1.424 (2)	C11—H11	0.9300
O4A—H4A	0.8200	C12—C13	1.499 (2)
O4A—H6D	0.4491	C13—H13A	0.9700
O8A—C13	1.399 (3)	C13—H13B	0.9700
O8A—H8A	0.8200	C13—H13C	0.9635
O8A—H13C	1.2178	C13—H13D	0.9733
O4B—C6	1.439 (5)	C14—C15	1.516 (2)
O4B—H4B	0.8200	C14—H14A	0.9700
O8B—C13	1.369 (13)	C14—H14B	0.9700
O8B—H8B	0.8200	C15—C16	1.517 (2)
C1—C2	1.337 (2)	C15—H15A	0.9700
C1—C6	1.503 (2)	C15—H15B	0.9700
C2—C3	1.428 (2)	C16—C17	1.504 (3)
C2—H2	0.9300	C16—H16A	0.9700
C3—C4	1.4541 (19)	C16—H16B	0.9700
C4—C5	1.3484 (19)	C17—H17A	0.9600
C5—C7	1.5073 (19)	C17—H17B	0.9600
C6—H6A	0.9700	C17—H17C	0.9600
			
C1—O1—C5	120.29 (11)	O6—C10—C11	124.42 (15)
C4—O3—H3	109.5	O6—C10—C9	120.11 (15)
C9—O5—H5	109.5	C11—C10—C9	115.47 (13)
C12—O7—C8	120.36 (12)	C12—C11—C10	120.66 (14)
C6—O4A—H4A	109.5	C12—C11—H11	119.7
H4A—O4A—H6D	104.7	C10—C11—H11	119.7
C13—O8A—H8A	109.5	C11—C12—O7	121.98 (14)
H8A—O8A—H13C	70.3	C11—C12—C13	127.48 (15)
C6—O4B—H4B	109.5	O7—C12—C13	110.50 (14)
C13—O8B—H8B	109.5	O8B—C13—O8A	52.8 (6)
C2—C1—O1	122.32 (13)	O8B—C13—C12	111.3 (6)
C2—C1—C6	126.71 (14)	O8A—C13—C12	115.07 (17)
O1—C1—C6	110.89 (13)	O8B—C13—H13A	140.2
C1—C2—C3	120.82 (13)	O8A—C13—H13A	108.5
C1—C2—H2	119.6	C12—C13—H13A	108.5
C3—C2—H2	119.6	O8B—C13—H13B	59.8
O2—C3—C2	124.45 (13)	O8A—C13—H13B	108.5
O2—C3—C4	120.46 (13)	C12—C13—H13B	108.5
C2—C3—C4	115.07 (12)	H13A—C13—H13B	107.5
O3—C4—C5	120.03 (12)	O8B—C13—H13C	108.9
O3—C4—C3	119.06 (12)	O8A—C13—H13C	58.6
C5—C4—C3	120.89 (13)	C12—C13—H13C	112.0
C4—C5—O1	120.60 (12)	H13A—C13—H13C	53.5
C4—C5—C7	126.41 (13)	H13B—C13—H13C	139.0
O1—C5—C7	112.99 (11)	O8B—C13—H13D	104.3
O4A—C6—O4B	109.7 (3)	O8A—C13—H13D	133.4
O4A—C6—C1	111.96 (15)	C12—C13—H13D	111.1
O4B—C6—C1	108.5 (2)	H13A—C13—H13D	60.5
O4A—C6—H6A	109.2	H13B—C13—H13D	48.6
C1—C6—H6A	109.2	H13C—C13—H13D	108.8
O4A—C6—H6B	109.2	C15—C14—C7	113.65 (12)
O4B—C6—H6B	108.1	C15—C14—H14A	108.8
C1—C6—H6B	109.2	C7—C14—H14A	108.8
H6A—C6—H6B	107.9	C15—C14—H14B	108.8
O4A—C6—H6C	109.0	C7—C14—H14B	108.8
O4B—C6—H6C	107.4	H14A—C14—H14B	107.7
C1—C6—H6C	110.2	C14—C15—C16	112.37 (14)
H6A—C6—H6C	107.1	C14—C15—H15A	109.1
O4B—C6—H6D	112.6	C16—C15—H15A	109.1
C1—C6—H6D	109.6	C14—C15—H15B	109.1
H6A—C6—H6D	112.1	C16—C15—H15B	109.1
H6B—C6—H6D	108.8	H15A—C15—H15B	107.9
H6C—C6—H6D	108.6	C17—C16—C15	113.87 (17)
C5—C7—C8	111.20 (12)	C17—C16—H16A	108.8
C5—C7—C14	112.71 (12)	C15—C16—H16A	108.8
C8—C7—C14	110.77 (11)	C17—C16—H16B	108.8
C5—C7—H7	107.3	C15—C16—H16B	108.8
C8—C7—H7	107.3	H16A—C16—H16B	107.7
C14—C7—H7	107.3	C16—C17—H17A	109.5
C9—C8—O7	120.75 (13)	C16—C17—H17B	109.5
C9—C8—C7	126.05 (13)	H17A—C17—H17B	109.5
O7—C8—C7	113.13 (12)	C16—C17—H17C	109.5
C8—C9—O5	119.76 (13)	H17A—C17—H17C	109.5
C8—C9—C10	120.76 (14)	H17B—C17—H17C	109.5
O5—C9—C10	119.48 (13)		
			
C5—O1—C1—C2	−0.8 (2)	C5—C7—C8—C9	118.54 (16)
C5—O1—C1—C6	176.06 (12)	C14—C7—C8—C9	−115.30 (16)
O1—C1—C2—C3	0.4 (2)	C5—C7—C8—O7	−64.36 (15)
C6—C1—C2—C3	−175.94 (14)	C14—C7—C8—O7	61.80 (15)
C1—C2—C3—O2	177.73 (16)	O7—C8—C9—O5	179.93 (13)
C1—C2—C3—C4	−0.6 (2)	C7—C8—C9—O5	−3.2 (2)
O2—C3—C4—O3	1.3 (2)	O7—C8—C9—C10	0.1 (2)
C2—C3—C4—O3	179.63 (13)	C7—C8—C9—C10	176.96 (13)
O2—C3—C4—C5	−177.25 (14)	C8—C9—C10—O6	179.85 (15)
C2—C3—C4—C5	1.1 (2)	O5—C9—C10—O6	0.0 (2)
O3—C4—C5—O1	179.94 (13)	C8—C9—C10—C11	0.5 (2)
C3—C4—C5—O1	−1.5 (2)	O5—C9—C10—C11	−179.41 (14)
O3—C4—C5—C7	0.1 (2)	O6—C10—C11—C12	−179.32 (16)
C3—C4—C5—C7	178.57 (13)	C9—C10—C11—C12	0.0 (2)
C1—O1—C5—C4	1.4 (2)	C10—C11—C12—O7	−1.1 (2)
C1—O1—C5—C7	−178.71 (12)	C10—C11—C12—C13	176.58 (17)
C2—C1—C6—O4A	−20.6 (2)	C8—O7—C12—C11	1.7 (2)
O1—C1—C6—O4A	162.71 (14)	C8—O7—C12—C13	−176.37 (14)
C2—C1—C6—O4B	100.7 (3)	C11—C12—C13—O8B	−29.2 (7)
O1—C1—C6—O4B	−76.1 (3)	O7—C12—C13—O8B	148.7 (7)
C4—C5—C7—C8	−111.66 (16)	C11—C12—C13—O8A	28.5 (3)
O1—C5—C7—C8	68.45 (15)	O7—C12—C13—O8A	−153.56 (17)
C4—C5—C7—C14	123.26 (16)	C5—C7—C14—C15	−61.92 (17)
O1—C5—C7—C14	−56.62 (16)	C8—C7—C14—C15	172.77 (13)
C12—O7—C8—C9	−1.1 (2)	C7—C14—C15—C16	−177.67 (14)
C12—O7—C8—C7	−178.40 (12)	C14—C15—C16—C17	178.09 (18)

### Hydrogen-bond geometry (Å, º)

**Table:**

*D*—H···*A*	*D*—H	H···*A*	*D*···*A*	*D*—H···*A*
O3—H3···O2^i^	0.82	1.96	2.7275 (15)	155
O3—H3···O2	0.82	2.32	2.7440 (15)	113
O5—H5···O6^ii^	0.82	2.00	2.7488 (16)	151
O5—H5···O6	0.82	2.32	2.7436 (16)	113
O4*A*—H4*A*···O2^iii^	0.82	2.07	2.839 (2)	157
O8*A*—H8*A*···O6^iii^	0.82	2.15	2.894 (2)	152

Symmetry codes: (i) −*x*, −*y*+1, −*z*+1; (ii) −*x*, −*y*+1, −*z*; (iii) *x*+1, *y*, *z*.
